# Epidemiology of Lyme borreliosis based on outpatient claims data of all people with statutory health insurance, Germany, 2019

**DOI:** 10.2807/1560-7917.ES.2022.27.32.2101193

**Published:** 2022-08-11

**Authors:** Manas K Akmatov, Jakob Holstiege, Lotte Dammertz, Joachim Heuer, Claudia Kohring, Martin Lotto-Batista, Friedrich Boeing, Stéphane Ghozzi, Stefanie Castell, Jörg Bätzing

**Affiliations:** 1 Department of Epidemiology and Health Care Atlas, Central Research Institute of Ambulatory Health Care, Berlin, Germany; 2 Department of Epidemiology, Helmholtz Centre for Infection Research, Braunschweig, Germany; 3 Department of Computational Hydrosystems, Helmholtz Centre for Environmental Research, Leipzig, Germany

**Keywords:** Lyme borreliosis, incidence, regional variations, claims data, notification data, Germany

## Abstract

**Introduction:**

Evidence of nationwide and regional morbidity of Lyme borreliosis (LB) in Germany is lacking.

**Aims:**

We calculated the total number of incident LB cases in Germany in 2019, compared regional variations, investigated the extent of possible under-reporting in notification data and examined the association between high incidence areas and land cover composition.

**Methods:**

We used outpatient claims data comprising information for people with statutory health insurance who visited a physician at least once between 2010 and 2019 in Germany (n = 71,411,504). The ICD-10 code A69.2 was used to identify incident LB patients. Spatial variations of LB were assessed by means of Global and Local Moran’s Index at district level. Notification data were obtained for nine federal states with mandatory notification from the Robert Koch Institute (RKI).

**Results:**

Of all insured, 128,177 were diagnosed with LB in 2019, corresponding to an incidence of 179 per 100,000 insured. The incidence varied across districts by a factor of 16 (range: 40–646 per 100,000). We identified four spatial clusters with high incidences. These clusters were associated with a significantly larger proportion of forests and agricultural areas than low incidence clusters. In 2019, 12,264 LB cases were reported to the RKI from nine federal states, while 69,623 patients with LB were found in claims data for those states. This difference varied considerably across districts.

**Conclusions:**

These findings serve as a solid basis for regionally tailored population-based intervention programmes and can support modelling studies assessing the development of LB epidemiology under various climate change scenarios.

## Introduction

Lyme borreliosis (LB) is the most frequent tick-borne disease in Europe, including Germany [1]. It is caused by spirochetes of the *Borrelia burgdorferi* sensu lato complex. The infection is transmitted through bites of the *Ixodes* tick species; *Ixodes ricinus* is the most frequent tick in Europe [2]. Increasing evidence suggests geographical expansion of LB in Europe, in particular into higher latitudes and altitudes [3]. The expansion is influenced by several factors, climate change being the most relevant [4]. The most common clinical manifestation of LB is erythema migrans, a localised skin lesion, occurring in more than 90% of all reported LB cases [5,6]. 

Lyme borreliosis is considered endemic in Germany [7]. Up to 20% of the German population become infected during their lifetime [8]. Notification of LB is mandatory in only nine of the 16 German federal states. Briefly, the six eastern federal states, Berlin, Brandenburg, Mecklenburg-Western Pomerania, Saxony, Saxony-Anhalt and Thuringia, introduced mandatory LB notification in 2001 [9]. Rhineland-Palatinate and Saarland, two western federal states, implemented mandatory notification in 2011 [10]. Since 2013, notification of LB has also been mandatory in Bavaria [5]. In these states covering 41% of the total German population, up to 14,000 LB cases are reported annually. The corresponding incidence ranged from 10 per 100,000 in 2013 to 16 per 100,000 persons in 2020. Evidence from the nine federal states suggests considerable regional variations in LB morbidity. Enkelmann et al. observed variations with estimates varying by a factor of 276 [10].

It is known that notification data suffer considerably from under-reporting [11]. This applies to LB in particular because of its often unspecific clinical course and, to a lesser extent, shortcomings of laboratory analysis in case of laboratory-based sentinel surveillance [1,12,13]. For example, research from the United States (US) estimated the absolute number of LB cases to be 10 times higher than the number of notified cases [14]. In accordance with these findings, several studies in Germany estimated much higher morbidity than recorded in the notification system. For example, a prospective population-based study in the region of Würzburg, Bavaria, reported an incidence of 111 per 100,000 individuals [6]. A considerably higher incidence was reported in another study that analysed nationwide claims data from one insurance company in Germany with 6 million insured, which corresponds to ca 7% of the total population: According to that study, the average annual incidence of LB amounted to 261 per 100,000 insured in 2007 and 2008 [15]. Although incidence estimates from these studies may not be directly comparable because of differences in case definition, it is commonly accepted that notification data underestimate the true morbidity of LB. Research has shown that the spread of LB is strongly linked to environmental factors. In particular, landscape characteristics were identified as risk factors for LB [16].

To provide more insights into the epidemiology of LB, we aimed to (i) calculate the total number of incident LB cases in Germany in 2019 based on the full sample of people with statutory health insurance, (ii) compare regional variations in LB morbidity, (iii) investigate the extent of possible under-reporting of LB in notification data and (iv) examine the association between high incidence areas and land cover composition. The latter three aims were examined at the Nomenclature of Territorial Units for Statistics level 3 (NUTS-3), i.e. district level.

## Methods

### Study population and data

We used nationwide outpatient claims data which comprise information about insured who visited an outpatient physician at least once between 2010 and 2019 (e.g. n = 71,411,504 in 2019). In brief, ca 87% of the total population had statutory health insurance in Germany in 2019. About 178,000 outpatient physicians, comprising general practitioners (GPs) and various medical specialists (e.g. neurologists, dermatologists) provided outpatient care in 2019. They are represented by the 17 regional Associations of Statutory Health Insurance Physicians (ASHIP). Physicians submit their claims for provided medical services to the patients’ health insurance fund through their ASHIPs and receive reimbursement according to the uniform medical fee scale. The data contained diagnoses, medical services provided by physicians (also in the context of outpatient surgery) as well as basic demographic characteristics of the insured such as sex, age (in years) and district of residence. The data did not contain information on inpatient treatment in hospitals. The outpatient physicians coded diagnoses according to the German modification of the 10th edition of the International Classification of Diseases and Related Health Problems (ICD-10-GM) [17]. In addition to the ICD-10 codes, they must apply modifiers such as ‘confirmed’, ‘suspected’, ‘status post’ or ‘excluded diagnosis’ to provide more diagnostic certainty.

### Case ascertainment

We defined patients as having incident LB if they received a diagnosis of Lyme disease (ICD-10 code A69.2) with a diagnostic modifier ‘confirmed’ for the first time in 2019. These patients needed to have a Lyme disease-free period between 2010 and 2018 to be considered as a new LB case.

### Notification data

In brief, nine federal states in Germany implemented mandatory notification for LB. Notifiable are the three most common clinical manifestations, erythema migrans, acute neuroborreliosis and Lyme arthritis, however, there are variations in case definitions of notifiable LB across federal states. In addition, in four federal states, notifications are done by physicians and diagnostic laboratories. In another four states, only physicians report LB cases, and one federal state implemented notification only by diagnostic laboratories. In all states, physicians and/or laboratories report LB cases to the local health authorities who further transmit the data to the health authorities at the federal level and finally to the Robert Koch Institute (RKI). We extracted notification data of LB for the year 2019 from the SurvStat database of the RKI [18]. The notification data, including absolute numbers of LB cases and incidence per 100,000 inhabitants were extracted in total as well as by district of residence (n = 209 districts). Data on the different manifestations of LB were not available.

### Land cover data

We obtained the CORINE land cover data at the NUTS-3 (district) level for the latest available year 2018 from the European Environmental Agency [19] and reclassified original land cover classes into nine relevant classes, comprising (i) non-green urban areas (this class includes urban fabric – which includes residential urban areas – industrial, commercial and transport units as well as mine, dump and construction sites), (ii) green urban areas (artificial, non-agricultural vegetated areas), (iii) green agricultural areas (agricultural areas except grassland), (iv) agricultural grassland (pastures), (v) forests, (vi) shrubs (shrubs and/or herbaceous vegetation associations), (vii) open spaces with little or no vegetation, (viii) wetlands and (ix) waterbodies. Land cover classes represented their relative proportion in each district. For example, the district Berlin consisted of 59.3% non-green urban areas, 17.8% forests, 12.0% green urban areas, 5.0% waterbodies, 2.5% green agricultural areas, 2.5% agricultural grassland, 0.7% shrubs and 0.1% wetlands.

### Statistical analysis

Initially, we calculated the incidence of LB per 100,000 insured by dividing the number of patients with the new diagnosis ‘Lyme disease’ in 2019 by the total number of insured in our database in the year. This analysis was also stratified by sex, age and region. Analysis of regional variations was done at the NUTS-3 level (n = 402 districts; administrative status on 31 December 2011). We used the Global Moran’s Index to examine the spatial autocorrelation of LB incidence [20]. In brief, the Moran’s Index is a global statistic that investigates spatial clustering in the whole region of interest (i.e. Germany). Its values range from −1 to +1 with negative values indicating the presence of districts with low incidences neighbouring districts with high incidences (i.e. outliers) and positive values pointing to the presence of districts with similar (low or high) incidences (i.e. clusters). Values close to zero indicate that the incidence of LB is distributed randomly, i.e. it does not show any spatial pattern in terms of its distribution. We defined neighbouring districts as those that have contiguous boundaries. If spatial clustering is present as indicated by the Global Moran’s Index, the Local Moran’s Index identifies the location and the size of spatial clusters in the region of interest [21]. There are four possible spatial cluster types: (i) clusters of districts with similar high incidence values (also called ‘hot spots’ or ‘high–high’), (ii) similar low values (‘cold spots’ or ‘low–low’), and dissimilar (i.e. (iii) high–low or (iv) low–high) incidence values (‘outliers’). In the remaining districts without statistical significance the incidence of LB is distributed randomly. 

Furthermore, we contrasted case numbers (in total) and incidence (in total and by district, n = 209) of LB from two data sources, outpatient claims and notification data. For both data sources, we calculated the 95% binomial confidence intervals according to Wilson [22] by using the total number of insured for claims data and population statistics obtained from the Federal Statistical Office for notification data [23]. Spearman’s rank correlation coefficient was used to examine the relationship of incidence in districts between claims and notification data. To compare the distribution and variation in incidence across districts we used natural log-transformed incidence. Finally, we examined the distribution of land cover data by two spatial clusters of the type low–low and high–high incidence. Mann–Whitney U test was used to test the differences in distribution between the two cluster types. All statistical analyses were performed with the R Foundation for Statistical Computing, version 3.3.2 [24].

## Results

In 2019, 71,411,504 individuals with statutory health insurance visited an outpatient physician at least once between 2010 and 2019. The proportion of females was higher in the study population with statutory health insurance than in the general population (Table). There were minor differences in the age distribution between the study and general population. Of note, the proportion of individuals older than 80 years was higher in the study population than in the general population.

**Table:** Representativeness of the study population^a^ in terms of sex, age and regional distribution, Germany, 2019 (n = 71,411,504)

Characteristics	Study population (n = 71,411,504)		General population (n = 83,166,711)	
	n	%	n	%
Sex				
Male	32,850,519	46.0	41,037,613	49.3
Female	38,560,985	54.0	42,129,098	50.7
Age group (years)				
0–9	6,895,463	9.7	7,688,346	9.2
10–19	6,285,614	8.8	7,642,156	9.2
20–29	8,341,141	11.7	9,682,902	11.6
30–39	9,269,765	13.0	10,784,930	13.0
40–49	8,372,641	11.7	10,182,384	12.2
50–59	11,188,250	15.7	13,447,540	16.2
60–69	8,810,706	12.3	10,506,803	12.6
70–79	6,582,465	9.2	7,550,515	9.1
≥ 80	5,665,459	7.9	5,681,135	6.8
Federal state				
Schleswig-Holstein	2,469,603	3.5	2,903,773	3.5
Hamburg	1,598,810	2.2	1,847,253	2.2
Bremen	598,471	0.8	681,202	0.8
Lower Saxony	6,961,035	9.7	7,993,608	9.6
North Rhine-Westphalia	15,664,208	21.9	17,947,221	21.6
Hesse	5,378,979	7.5	6,288,080	7.6
Rhineland-Palatinate	3,402,166	4.8	4,093,903	4.9
Baden-Württemberg	9,158,243	12.8	11,100,394	13.3
Bavaria	10,994,475	15.4	13,124,737	15.8
Berlin	3,117,459	4.4	3,669,491	4.4
Saarland	849,449	1.2	986,887	1.2
Mecklenburg-Western Pomerania	1,441,848	2.0	1,608,138	1.9
Brandenburg	2,202,883	3.1	2,521,893	3.0
Saxony-Anhalt	2,006,939	2.8	2,194,782	2.6
Thuringia	1,913,320	2.7	2,133,378	2.6
Saxony	3,653,616	5.1	4,071,971	4.9

^a^ Data for the general German population originate from the Federal Statistical Office [23].

**Figure 1:**
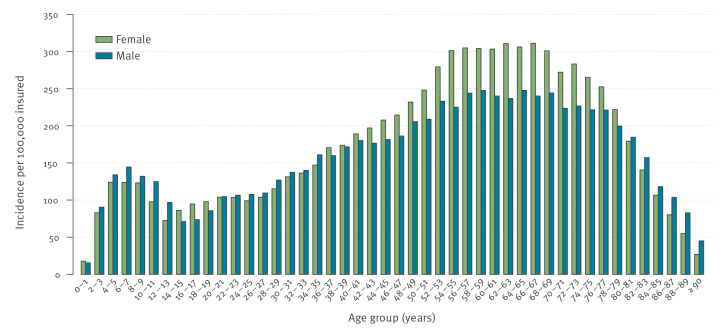
Incidence of Lyme borreliosis by sex and age based on outpatient claims data, Germany, 2019 (n = 71,411,504)

Of the 71,411,504 insured, 128,177 were newly diagnosed with LB in 2019, an incidence of diagnosed LB of 179 per 100,000 insured. Of these, 55,090 (43%) were male and 73,087 (57%) were female. In terms of age distribution, we observed a bimodal distribution (Figure 1). The first incidence increase was among 6–7-year-old male children with an incidence of 144 per 100,000 and 4–9-year-old female children with an incidence of 124 per 100,000 insured. The second increase was among older age groups with the highest incidence among 64–65-year-old males and 66–67-year-old females (Figure 1).

### District-level variations

**Figure 2:**
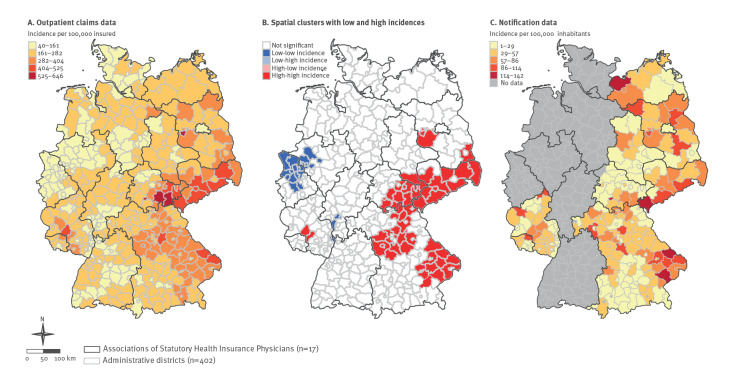
Geographical distribution of the incidence of Lyme borreliosis based on notification and outpatient claims data and spatial clusters with low and high incidences, on the level of administrative districts, Germany, 2019

The incidence of LB based on outpatient claims data varied across districts by a factor of 16 with the lowest incidence of 40 per 100,000 in Oberhausen (North Rhine-Westphalia) and the highest in Brandenburg an der Havel (Brandenburg) with 646 per 100,000 insured. The incidences were highest in districts of three eastern federal states, Brandenburg, Saxony and Thuringia and two western federal states, Bavaria and Rhineland-Palatinate (Figure 2A). The spatial distribution of LB across districts was not random as indicated by the Global Moran’s I test (I = 0.65, p < 0.0001). The Local Moran’s I test showed the presence of four spatial clusters with high incidences containing 55 districts in total (Figure 2B). The biggest spatial cluster comprising 40 districts from five federal states (including four eastern federal states, Brandenburg, Saxony, Saxony-Anhalt and Thuringia and one western federal state, Bavaria) was observed in the south-east part of Germany. The second largest spatial cluster was located in the east of Bavaria. This cluster incorporated 11 districts located in the Bavarian forest. Two smaller spatial clusters were located in the federal states Brandenburg (n = 2 districts) and Rhineland–Palatinate (n = 2 districts).

Incidences are classified in five groups with equal intervals. Panel A: study population of 71,411,504 insured. Panel B: local clusters with two spatial patterns were identified by Local Moran’s I test. Districts with low–high and high–low spatial patterns were not observed. All remaining districts (white colour) did not show any spatial pattern in terms of LB distribution. Panel C: 209 districts from nine federal states with mandatory notification [18]. In districts without data (grey colour) there is no mandatory notification.

There are 17 regional ASHIPs in Germany, of which 15 coincided with 15 German federal states. Two ASHIPs exist in the federal state of North Rhine-Westphalia.

### Incidence based on notification and claims data

**Figure 3:**
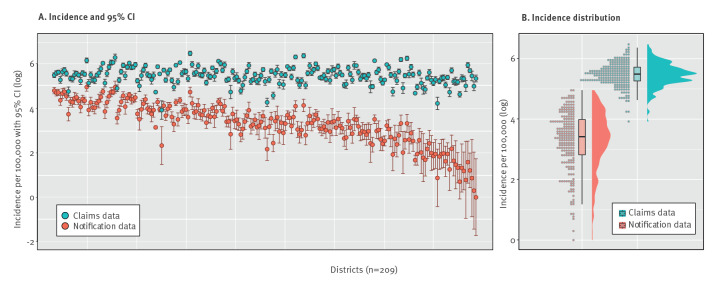
Incidence of Lyme borreliosis at district level based on notification and outpatient claims data, Germany, 2019 (n = 209 administrative districts)

**Figure 4:**
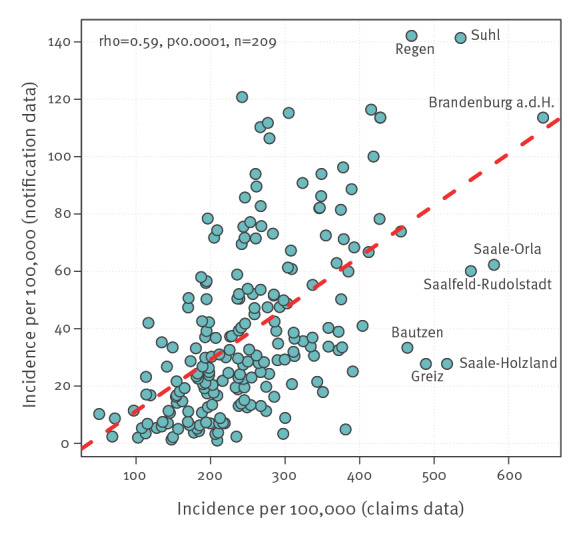
Scatter plot depicting the incidence of Lyme borreliosis at district level based on notification and claims data, Germany, 2019 (n = 209 administrative districts)

In 2019, 12,264 LB cases were reported to the RKI from nine federal states with mandatory notification. In the same period of time and in the same federal states, claims data contained 69,623 insured who were newly diagnosed with LB, a factor of 5.7 of relative difference between the two data sources. The corresponding incidences in notification and claims data were 15 per 100,000 and 235 per 100,000 persons, respectively. At district level, the incidence of LB in notification data varied strongly between 1 per 100,000 in the district of Miesbach and 142 per 100,000 in the district of Regen (both Bavaria) (Figure 2C and 3A). Notably, the incidence of LB was higher in all districts based on claims than notification data, however, to a varying extent. The relative differences in incidence between districts based on notification and claims data varied by factors between 2.4 and 209 (median: 8.1). Variation in the incidence of LB across districts based on claims data was less pronounced than based on notification data (Figure 3B). The incidence of LB in both data sources displayed a moderate correlation (Spearman’s rho = 0.59; p < 0.0001) (Figure 4).

CI: confidence interval.

Panel A: Each circle represents a district. Districts are sorted in ascending order by the magnitude of the relative differences in incidence between notification and claims data. Notification data are freely accessible from [18].

Each circle represents a district. Notification data are freely accessible from [18]. The Spearman’s rank correlation coefficient (rho) was used to examine the relationship of the incidence values from two data sources.

### Association with land cover classes

**Figure 5:**
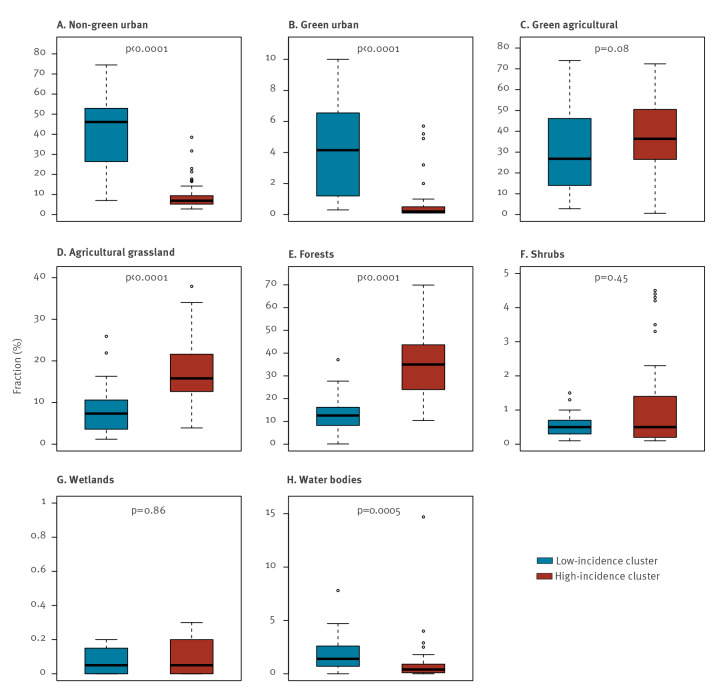
Boxplots depicting the distribution of land cover classes at district level by Lyme borreliosis spatial incidence cluster type, Germany, 2019

We examined whether the land cover composition of the districts differed between two cluster types (low–low and high–high incidence of LB). Of nine reclassified land cover classes, the class ‘open spaces with little or no vegetation’ was excluded from the analysis as there were no districts with this type of land cover in the low–low incidence cluster. Also, there were only five districts with this land cover class in the spatial cluster high–high. Of the remaining eight land cover classes, we observed statistically significant differences between the two spatial cluster types in five land cover classes (Figure 5). We found clear evidence of association: districts with a higher proportion of forests and agricultural grasslands were significantly more frequent in high-incidence clusters, whereas urban areas were more represented in low-incidence clusters.

Spatial clusters of the type low–low (n = 28 districts) and high–high (n = 55 districts) incidence were identified by Local Moran’s I test (see Figure 2B). The Mann–Whitney U test was used to examine the differences in various land cover classes by two spatial cluster types.

## Discussion

Using recent nationwide outpatient claims data, we described the epidemiological situation regarding LB in Germany. In particular, we provided for the first time nationwide and regional morbidity estimates and examined the extent of possible under-reporting in notification data at a small-area scale. These findings are of considerable importance since data on epidemiology of LB in Germany are scarce [7]. Evidence from previous research yields heterogeneous findings regarding the frequency of LB with incidence values ranging widely between 18 and 280 per 100,000 population. Notification data from the nine federal states report the lowest incidences, highlighting possible under-reporting of LB [5,10,25,26]. An early study by Fulop and Poggensee reported an incidence of 18 per 100,000 persons in 2002 and a later incidence of 37 per 100,000 persons in 2006 [25]. Several years later, the incidence remained similar between 26 per 100,000 in 2015 and 41 per 100,000 in 2013 without a clear time trend [10]. Finally, an incidence of 47 per 100,000 was observed in the federal state of Bavaria in 2020 [5]. However, it is well known that notification data underestimate the true morbidity as they suffer from issues such as under-reporting and misclassification [27]. According to national data, ca 85,000 LB cases are reported annually in Europe [28]. However, the actual number of LB cases is assumed to be much higher. Another study estimated that ca 232,000 cases occur annually in Europe [29]. Also, a study in the United Kingdom showed that the number of LB cases was three times higher than previously reported estimates [30]. Under-reporting of LB is assumed to occur also outside Europe. For example, Nelson et al. estimated that around 329,000 LB cases occur each year in the US, compared with 30,000 cases recorded in the notification system. Thus, this figure was more than 10 times higher than the number of notified cases [31]. Our observation was in agreement with these findings, although the difference between notification and claims data was smaller than in the studies above. The number of LB cases in claims data amounted to ca 69,000 vs 12,000 notified cases, a nearly six-fold difference. Primary and secondary data studies in Germany showed morbidity estimates much higher than that of notification data [6,15]. Our incidence value of 179 per 100,000 insured is also higher than the estimate from notification data (14.8 per 100,000 persons) and lies between estimates from primary (111 per 100,000 persons) [6] and secondary data studies (279 per 100,000 persons) [15]. Of note, we used the full sample of all people with statutory health insurance, covering 87% of the total German population. Thus, findings of the present study may be considered highly representative from a population point of view.

Several limitations of the study should be mentioned. Firstly, medical claims data were primarily collected by physicians for billing purposes and not for epidemiological research. Diagnostic codes, including the diagnosis of LB have not been validated and may thus result in misclassification. Both under- and overdiagnosis in claims data have been reported. For example, Erler et al. compared claims data with patients’ medical records from GPs and observed that the rates of under- and overdiagnosis were 30% and 20%, respectively [32]. Although we used a conservative case definition (i.e. a confirmed diagnosis of LB rather than e.g. suspected diagnosis), the risk of misclassification cannot be ruled out. Factors such as physicians’ coding behaviour and patients’ help-seeking behaviour may further influence the validity of claims data. For example, there is only a general definition for using diagnostic modifiers such as confirmed or suspected diagnosis. However, disease-specific definitions are not yet implemented and the final decision on its use remains the individual responsibility of physicians, which may vary regionally and result in false associations in terms of geographical variations. Secondly, because clinical manifestations of LB are often unspecific and uncommon, many LB cases may remain undetected. Thus, we analysed mainly symptomatic patients. However, this limitation should apply equally to notification data, possibly even to a greater extent. Thirdly, case definitions applied in notification and claims data may not be directly comparable. Fourthly, we excluded those patients who were diagnosed with LB in the period between 2010 and 2018 and thus ignored a possible reinfection. This may result in underestimation of the incidence. Fifthly, another possible limitation of the study is the existence of the GP-centred healthcare in some parts of Germany (e.g. Baden-Württemberg and Bavaria). The GPs providing this type of healthcare are still members of a regional ASHIP but submit their claims directly to health insurance companies. Thus, data from this type of healthcare are not included in our dataset. Thus, the morbidity estimates in these regions may be underestimated, which may limit the interpretation of regional findings. The extent of this limitation is unknown. Sixthly, we used the patients’ district of residence for regional analysis. The actual district of tick exposure is unknown and may differ from the district of residence. However, Enkelmann et al. observed that overwhelming majority of LB infections (90%) occur at the district of residence [10]. Finally, our dataset does not contain information of privately ensured individuals who account for 11–12% of the German population. Individuals with private health insurance have a higher socioeconomic status than those with statutory health insurance and may differ in terms of infection risk for LB, although data supporting this are not available. Unfortunately, further information such as education level, income or profession was not available in our dataset.

Nevertheless, several of our findings support the validity of claims data. A very similar sex and age distribution of LB morbidity was observed in other studies that analysed notification data from nine federal states [10,26]. In terms of age, Enkelmann et al. reported a bimodal distribution with two peaks, at the age group of 5–9 years and 60–69 years. Similar findings were also reported by Böhmer et al. who analysed notification data from one federal state [5]. The typical bimodal distribution was also observed in studies from other countries (e.g. in England and Wales) [33]. Furthermore, the incidence at district level based on claims correlated well with that based on notification data [5,10]. Thus, although notification data may underestimate the incidence of LB, they can be used to monitor epidemiological patterns, including temporal and regional variations.

Little is known about regional variations of LB morbidity in the whole of Germany. Previous studies demonstrated differences for rough geographical areas [9]. Enkelmann et al. observed very strong variations in the incidence of notified LB across districts varying between 0.5 and 138 per 100,000 in the years 2013 to 2017 [10]. To date, geographical variations in regions without mandatory notification of LB are unknown. To close this gap, we applied spatial analysis using the Germany-wide data. We found four spatial clusters in Germany, some of them roughly coinciding with the risk regions identified by Enkelmann et al. [10]. To further investigate the spatial clusters, we compared land cover composition between low- and high-incidence clusters. The very clear associations we find, in line with the literature [34], further support the relevance of claims data for Lyme disease epidemiology and call for further studies of links between disease spread and environment, including weather and climate conditions. Other findings that can be considered as indirect evidence of under-reporting of LB in notification data. Firstly, the incidence of LB in all districts was higher based on claims than notification data. However, incidences in districts from both data sources showed a similar geographical pattern. Secondly, the regional variation observed in claims data was less pronounced than that from notification data. This can be explained by a less specific case definition of LB in claims data which results in lesser variations across districts. However, larger variation in notification data may also be explained by under-reporting, which varies regionally with some regions having a well implemented notification system and/or better compliance among physicians than in other regions. In addition, there are variations in the reporting system across the federal states, e.g. physician/diagnostic laboratories, name-based/anonymous notification or different case definitions. Thirdly, the incidence of LB based on claims data followed a nearly normal distribution, whereas notification data exhibited a right-skewed form with a considerable number of districts with low incidence values. Our findings are of particular importance for local public health authorities to improve their notification systems. In addition, these results show the relevance of using reliable data sources for understanding drivers of disease at different scales. Since there are no nationwide data on LB in Germany, the medical claims data serve as a reliable and more resource-efficient alternative source to the existing notification data to monitor epidemiological trends. Other European countries implemented various approaches for monitoring the epidemiology of LB (e.g. a physician sentinel network in Switzerland [35] or a combination of laboratory sentinel and hospitalisation data in Belgium [36]). Considering that there is a non-random spatial distribution of LB cases in the country, quantitative methods including multiple covariates would allow to understand and quantify potential drivers of disease in Germany. The findings of the study may also be of interest for other European countries with similar environmental conditions.

## Conclusions

We observed considerable regional variations in the distribution of LB, with some districts identified as high-risk regions for LB. The largest spatial cluster comprised 40 districts spanning across several federal states. Notably, Germany as a whole should be considered endemic because LB was observed in all districts. Moreover, we found clear associations with land cover composition, which show that the analyses at the district level are fine enough to account for environmental factors. The findings may serve as a basis for regionally tailored population-based preventive programmes and can support modelling of LB under various climate change scenarios. This is of particular importance as there is no licensed vaccine against LB in Germany. Preventive measures to increase of awareness of LB prevention should take place in regions with high LB morbidity.
